# Simulation of the Metabolism of New Psychoactive Substances Using Electrochemistry‐Mass Spectrometry: Introducing an Innovative Software Tool for Rapid Data Evaluation

**DOI:** 10.1002/dta.70006

**Published:** 2025-11-27

**Authors:** Mark Wesner, Steffen Heuckeroth, Michael Pütz, Uwe Karst

**Affiliations:** ^1^ Institute of Inorganic and Analytical Chemistry University of Münster Münster Germany; ^2^ Federal Criminal Police Office Forensic Science Institute Wiesbaden Germany

**Keywords:** electrochemistry, mass spectrometry, MDMB‐4en‐PINACA, mzmine, new psychoactive substances

## Abstract

An innovative software tool for the rapid and efficient simulation of the metabolism of new psychoactive substances (NPS) was developed, based on the open‐source project mzmine, and applied. NPS are compounds designed to mimic the psychotropic effects of established illicit drugs while circumventing drug legislation. These compounds are developed solely regarding their desired effects, thus possibly leading to harmful side effects including the formation of toxic metabolites. Analytical reference standards, needed to carry out metabolic studies, are not immediately available because emerging NPS are primarily discovered subsequent to drug confiscations. Using these confiscated substances in traditional metabolic in vivo or in vitro studies is often not possible due to the substances being impure or being a part of a mixture of different NPS. Therefore, a software tool was developed to streamline the evaluation of data acquired by the online combination of electrochemistry and mass spectrometry for the simulation of NPS metabolism. Using this tool, it is possible to generate mass voltammograms directly from mass spectrometric raw data. Combining this newly implemented tool with existing filtering algorithms in mzmine, we simulated the metabolism of the synthetic cannabinoid receptor agonist (SCRA) methyl 3,3‐dimethyl‐2‐[1‐(pent‐4‐en‐1‐yl)‐1*H*‐indazole‐3‐carboxamido] butanoate (MDMB‐4en‐PINACA) from a mixed solution of different NPS. Fragmentation data indicated that one of the transformation products found for MDMB‐4en‐PINACA is likely of a quinoid structure. The potential formation of this possibly highly reactive quinoid metabolite could be a first hint for possible causes of adverse side effects frequently reported after the recreational use of MDMB‐4en‐PINACA and related SCRAs.

## Graphical abstract

A novel software tool for the rapid and efficient simulation of the metabolism of new psychoactive substances was developed and applied to mimic the metabolism of the synthetic cannabinoid receptor agonist MDMB‐4en‐PINACA.

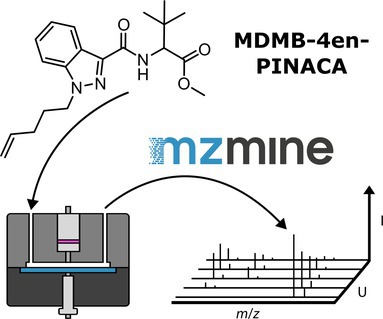

## 1 Introduction

New psychoactive substances (NPS), being represented in various substance classes with different psychotropic properties, are specifically designed or selected to mimic the effects of established illicit drugs while circumventing drug legislation [1]. Currently, 1000 different compounds are monitored by the European Drug Agency (EUDA, former European Monitoring Centre on Drugs and Drug Addiction) [2]. While some of those substances represent drug candidates rejected in clinical trials, many substances are specifically designed for the drug market without any prior metabolic studies being carried out, resulting in poor knowledge of their metabolism and toxicity [3]. Therefore, adverse side effects are frequently observed after recreational use of NPS [4]. Novel NPS are primarily discovered subsequent to drug confiscations where seized substances are often impure, adulterated, or mixtures of different NPS [5, 6]. Their direct use for traditional metabolic in vivo and in vitro studies is often not possible, as rather laborious and time‐consuming purification steps are needed. Together, this drastically slows down the elucidation of the metabolism of novel NPS. Hence, the identification of potentially toxic metabolites is delayed. Additionally, the possibility for forensic‐toxicological investigations regarding an individual's NPS consumption as well as the determination of NPS consumption in the population are affected negatively. Here, for both urine analysis in forensic use cases and estimation of NPS consumption in large populations by wastewater‐based epidemiology, precise knowledge of the metabolism of a substance is crucial [7]. To streamline and accelerate the time‐consuming process of gathering the needed analytical data on novel NPS, the EU‐project ADEBAR, a competence network of nine forensic laboratories from police, customs, and several universities, has been established in 2017 in Germany. The project aims to comprehensively and rapidly characterize newly surfacing psychotropic substances relevant for forensic‐toxicological casework and to distribute the analytical datasets digitally through European and (inter)national channels [8].

**FIGURE 1:**
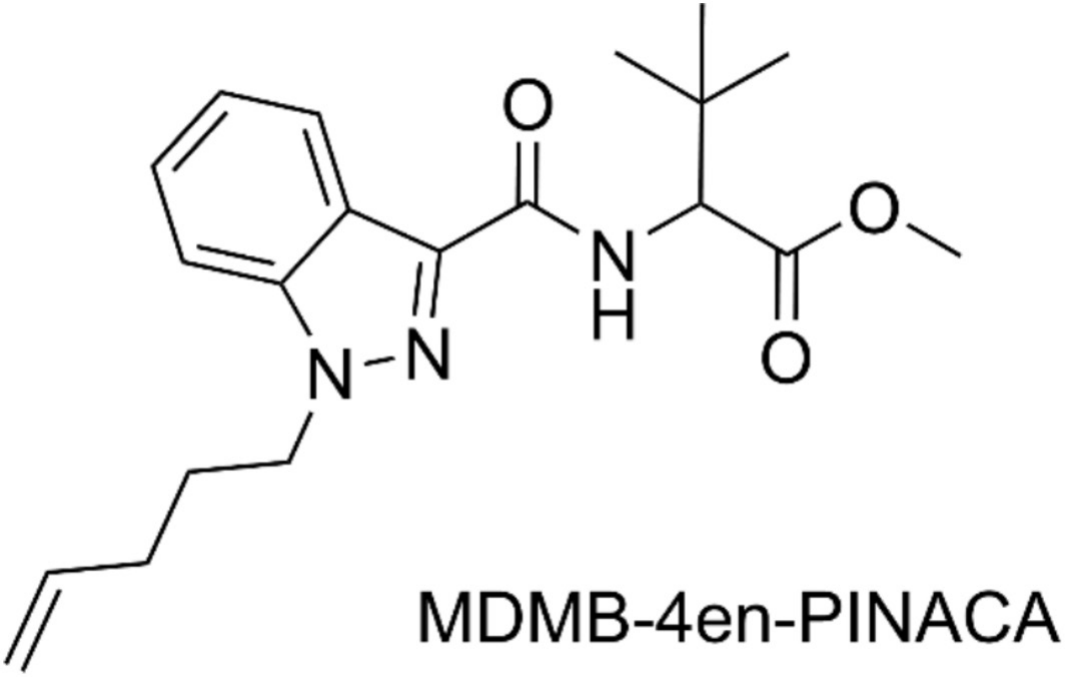
Structural formula of the new psychoactive substance MDMB‐4en‐PINACA.

MDMB‐4en‐PINACA (methyl 3,3‐dimethyl‐2‐[1‐(pent‐4‐en‐1‐yl)‐1*H*‐indazole‐3‐carboxamido] butanoate, Figure 1) is a NPS that mimics the psychotropic effects of Δ [9]‐tetrahydrocannabinol (THC), the main active ingredient in cannabis. It therefore belongs to the class of synthetic cannabinoid receptor agonists (SCRA), the largest subclass of NPS with 274 different compounds being monitored by the EUDA by the end of 2024 [2]. SCRAs were first identified in herbal blends sold under the name “Spice” in 2008 by Auwärter et al. [9] In 2017, MDMB‐4en‐PINACA was first reported in Europe and gained traction quickly [10]. By 2020, it was one of the most commonly detected SCRAs in many European countries including Germany, where a total of 2667 seizures of material containing MDMB‐4en‐PINACA were reported by German police and customs authorities from 2017 until February 2024 [11]. MDMB‐4en‐PINACA was the substance most frequently identified in low‐THC cannabis flowers adulterated with SCRAs. These pose a severe health risk, as the adulterated plant material cannot be distinguished from regular cannabis flower by visual or olfactory inspection [12, 13]. Cannabis users may therefore be unaware of consuming SCRAs, which often pose a greater risk of adverse and even toxic effects when compared to regular cannabis [14, 15]. Additionally, MDMB‐4en‐PINACA was one of the most prevalent SCRAs detected on impregnated paper sheets, frequently smuggled into prisons, causing multiple problems including severe intoxications and outbreaks of violence among prisoners. [16, 17]

In contrast to traditional metabolic studies like in vivo rat or in vitro cell studies, electrochemistry (EC) hyphenated online to mass spectrometry (MS) is a complementary and purely instrumental approach to metabolism simulation [18, 19, 20, 21]. It has been shown that many reactions catalyzed by enzymes of the Cytochrome P450 family, like hydroxylations and dealkylations, can be simulated by EC‐MS making it a valid approach to the simulation of drug metabolism [22, 23]. Furthermore, the well‐controlled chemical environment in combination with the rapid identification by MS enables the detection even of reactive transformation products (TPs), which would not be accessible through traditional approaches for the simulation of the metabolism of drugs [19, 20, 24]. Combined with high‐resolution tandem mass spectrometry (HRMS/MS) for further characterization of generated TPs, EC‐MS is therefore a well‐suited tool for rapid screening of possible metabolites for a vast amount of different compounds [18, 19, 20, 23, 25, 26].

In this work, a novel software tool, facilitating the processing and evaluation of data acquired by EC‐MS experiments, is developed based on the open source project mzmine [27]. The newly developed tool is showcased studying the oxidative behavior of the SCRA MDMB‐4en‐PINACA, which was selected as a model substance because of its high consumption prevalence, especially in Germany [2, 11, 13, 17]. To mimic a real‐life case of mixtures of NPS being investigated, the newly developed tool is combined with filtering algorithms in mzmine to simulate the metabolism of MDMB‐4en‐PINACA directly from a mixed solution of different SCRAs.

## 2 Materials and Methods

### 2.1 Chemicals

Ammonium formate (99%) and ammonia (25% solution, Ph. Eur.) were obtained from Sigma‐Aldrich Chemie (Steinheim, Germany). HPLC‐MS grade acetonitrile (ACN) was purchased from VWR Chemicals (Darmstadt, Germany). The SCRAs MDMB‐4en‐PINACA (93.4%) and (S)‐*N*‐(1‐amino‐1‐oxo‐3‐phenylpropan‐2‐yl)‐1‐(5‐fluoropentyl)‐1*H*‐indazole‐3‐carboxamide (PX‐2, 98.3%) were supplied by the Federal Criminal Police Office (Wiesbaden, Germany). Water was purified using a Milli‐Q EQ 7000 ultrapure water system (MerckMillipore, Darmstadt, Germany).

### 2.2 Electrochemical Oxidation of Synthetic Cannabinoid Receptor Agonists

The SCRAs were oxidized using an electrochemical thin‐layer cell (μ‐PrepCell 2.0 by Antec Scientific, Alphen aan den Rijn, Netherlands). The cell made use of a three‐electrode setup consisting of a Pd/H_2_ pseudo reference electrode (pRE), a boron‐doped diamond working electrode and a conductive polyether ether ketone counter electrode. Potentiostatic experiments were carried out at room temperature.

The electrolyte consisted of a 20‐mM aqueous ammonium formate solution adjusted with ammonia to a pH of 7.4 and ACN (50/50; *v*/*v*). Two solutions of the SCRAs were prepared in this electrolyte. The first solution contained solely PX‐2 in a concentration of 1 μM; the second solution contained both PX‐2 and MDMB‐4en‐PINACA in a concentration of 1 μM each. Simulation of the metabolism was carried out by online EC‐HRMS/MS by perfusing the solutions through the electrochemical cell at a flow rate of 20 μL min^−1^ using a syringe pump and transferring the effluent directly to the mass spectrometer using a capillary with an inner volume of 10 μL. A potential ramp from 0 to 3500 mV vs. Pd/H_2_ pRE with a scan rate of 10 mV s^−1^ and a step size of 5 mV was applied to the cell. The potentiostat as well as the software used to control the potentiostat were developed and built in‐house. The utilized trapped ion mobility spectrometry time‐of‐flight mass spectrometer was a timsTOF fleX controlled by timsControl 5.0.9 (both Bruker Daltonics, Bremen, Germany). The mass spectrometer was operated in positive electrospray ionization mode. HRMS/MS spectra were acquired in data‐dependent acquisition (DDA) mode. Detailed mass spectrometric parameters are provided in the Supporting Information (Tables S1 and S2). The open‐source software mzmine 3.6.0 [27] was used for data processing as well as data evaluation and exporting of mass voltammograms. Exported mass voltammograms were redrawn in Origin 2021b (OriginLab Corporation, Northampton, MA, USA) to enhance the plots' visual appearance for publishing. The resulting graphs, the so‐called mass voltammograms, were used to visualize the electrochemical transformation process.

## 3 Results and Discussion

### 3.1 Novel Software Tool

The open‐source project mzmine [27] was used as a foundation for the development of a novel software tool for processing and evaluation of data acquired by EC–MS experiments. The tool is able to visualize the acquired data in the form of a mass voltammogram directly from the mass spectrometric raw data file without the need for any previous data processing steps. It is fully integrated into the mzmine ecosystem and accessible to all users starting from release 3.3.0 resulting in the previous application of the software tool in studies facilitating their data evaluation workflow [25, 26].

**FIGURE 2:**
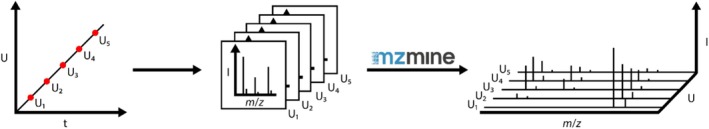
Schematic overview on the generation process of mass voltammograms in mzmine.

Figure 2 depicts an overview on the generation of mass voltammograms using mzmine. The user imports the raw data file and sets the parameters of the corresponding potential ramp. Based on this information, the software tool extracts mass spectra from the imported raw data file and plots them in a three‐dimensional waterfall diagram against the corresponding applied potentials, resulting in the mass voltammogram. Instead of generating the mass voltammogram as a static two‐dimensional figure, it is rather generated as a three‐dimensional object. The mass voltammogram can be scaled and rotated by the user in real time. Furthermore, it is also possible to annotate all signals with their mass‐to‐charge‐ratio (*m*/*z*), as well as their intensity and formation potential. Together, this significantly accelerates the evaluation of acquired data. For further use, it is possible to export the mass voltammogram directly into several common image file formats. Additionally, the raw data visualized in the mass voltammogram can be exported into different tabular data file formats such as the CSV and XLSX file formats. This opens up the possibility to plot the mass voltammogram in any other graphing software.

Utilizing mzmine also positively benefits the possibilities of processing the EC‐MS data. Once the data are imported, it can be processed by making use of the full capabilities of data workflows in mzmine. Mass voltammograms can be generated from the processed data afterwards. Especially useful built‐in data processing features in mzmine are, for example, the possibility to perform a blank subtraction to eliminate background interferences or the grouping of isotope signals [28, 29]. Using these features, it is possible to greatly reduce the spectral complexity of the mass voltammogram.

### 3.2 Metabolism Simulation

**FIGURE 3:**
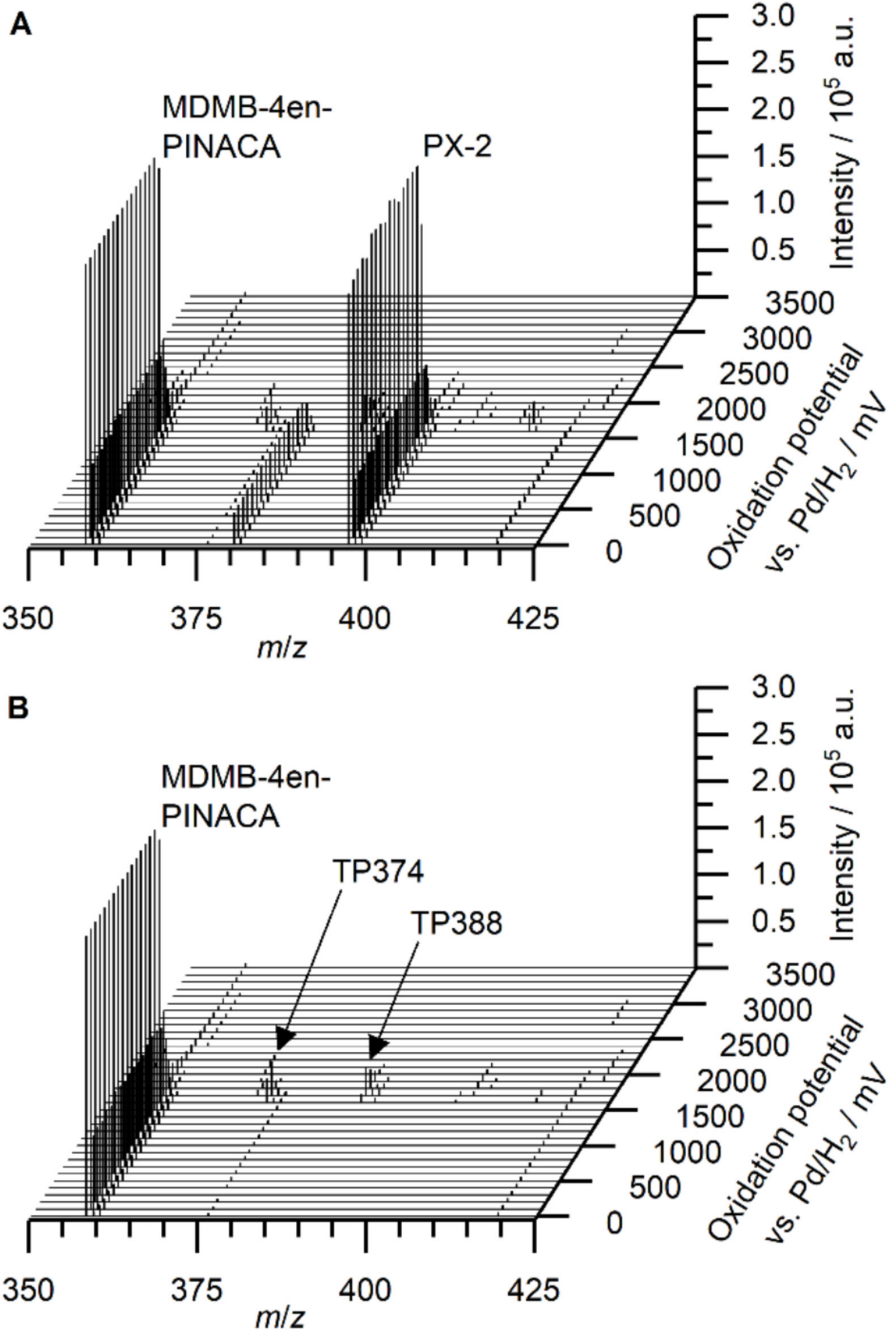
Mass voltammograms for the electrochemical oxidation of a mixture of MDMB‐4en‐PINACA and PX‐2 between 0 and 3500 mV on a boron‐doped diamond working electrode before (A) and after subtraction of signals corresponding to PX‐2 (B).

To demonstrate the generation of mass voltammograms from processed feature lists, the oxidation behavior of the SCRA MDMB‐4en‐PINACA was studied using a mixed standard solution. The solution contained the analyte of interest, MDMB‐4en‐PINACA, alongside another SCRA, namely, PX‐2. Figure 3A depicts the resulting raw data in the form of a mass voltammogram generated by the newly developed software tool introduced above. Two intense signals can be observed that both decrease in intensity with increasing applied oxidation potential. Those signals can be assigned by their *m*/*z* to the protonated species of the analyte of interest, MDMB‐4en‐PINACA, as well as to the simulated interference, PX‐2. Additionally, several less intense signals can be observed that form at different potentials, therefore corresponding to different TPs. With both MDMB‐4en‐PINACA and PX‐2 being oxidized simultaneously in the electrochemical cell, it is not immediately possible to assign the TPs to their corresponding parent compound.

Here, the possibility to process the data by making use of the built‐in data workflows in mzmine comes in beneficially. As a reference, a second solution containing solely PX‐2 was oxidized under the same conditions as the mixed standard solution. By subtracting the signals from this reference solution from the data acquired for the mixed standard solution, only signals corresponding to MDMB‐4en‐PINACA remain. Due to the synergistic interplay between the newly developed software module and the established mzmine software platform, it is possible to generate a mass voltammogram from the resulting data after subtraction. Using this workflow, a mass voltammogram for MDMB‐4en‐PINACA can be generated from the mixed standard solution without the need for a pure analytical standard of MDMB‐4en‐PINACA. This approach can also be applied when a novel NPS is discovered as part of a mixture of other, already established NPS. Because there are typically reference standards available for the established NPS, the NPS mixture can be directly used for the simulation of the metabolism of the newly discovered NPS. A previous purification of the NPS mixture would not be necessary as long as analytical reference standards are available for all adulterants present in the mixture. Although, this approach could be limited by the complexity of the NPS mixture. Here, the accuracy of the background subtraction in cases of mixtures of multiple structurally similar compounds needs to be further addressed in the future. Simultaneous electrochemical transformation of structurally similar compounds could result in interfering TPs possibly impairing the background subtraction process.

Figure 3B depicts the resulting mass voltammogram where only signals corresponding to the analyte of interest, MDMB‐4en‐PINACA, and its TPs are observed. The signals corresponding to PX‐2 were removed. The two major TPs are both formed at a potential of around 1700 mV vs. Pd/H_2_ pRE. According to their *m*/*z* of 374.2078 and 388.1873, respectively, they are referred to as TP374 and TP388. Detected accurate *m*/*z*, proposed ion formulae, exact *m*/*z*, and resulting mass deviations (Δ*m*/*z*) are summarized in Table 1.

**TABLE 1:** Recorded *m*/*z*, assigned ion formulae, calculated *m*/*z*, and resulting mass deviations (Δ*m*/*z*) for MDMB‐4en‐PINACA and the main TPs detected during online EC‐HRMS/MS analysis.

Compound	Recorded *m*/*z*	Ion formula	Calculated *m*/*z*	Δ*m*/*z*/ppm
MDMB‐4en‐PINACA	358.2129	C_20_H_28_N_3_O_3_^+^	358.2125	1.1
TP374	374.2078	C_20_H_28_N_3_O_4_^+^	374.2074	1.1
TP388	388.1873	C_20_H_26_N_3_O_5_^+^	388.1867	1.5

For TP374, a mass difference of 15.9949 Da to MDMB‐4en‐PINACA was observed. This, together with the predicted ion formula shown in Table 1, suggests that TP374 corresponds to a hydroxylated product of MDMB‐4en‐PINACA. Accordingly, for TP388, a mass difference of 29.9744 Da to MDMB‐4en‐PINACA was observed. The addition of two oxygen atoms as well as the loss of two hydrogen atoms result in a theoretical mass difference of 29.9742 Da. This suggests that TP388 corresponds to a doubly hydroxylated and dehydrogenated product of MDMB‐4en‐PINACA.

**FIGURE 4:**
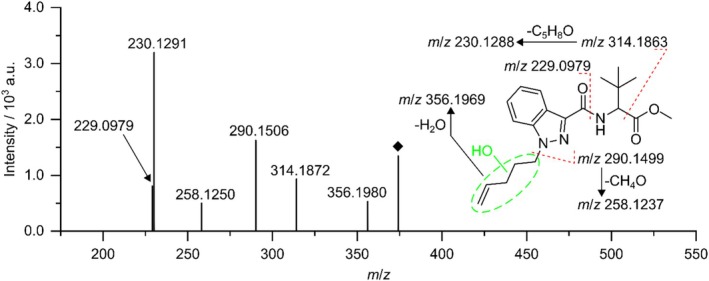
HRMS/MS spectrum and proposed fragmentation pattern of TP374. Changes in the molecular structure introduced during the electrochemical oxidation are highlighted in green. The acquisition of fragment spectra was carried out alongside the mass voltammogram using DDA. The spectrum was obtained by summation of HRMS/MS spectra with collision energies of 15 and 30 eV, respectively.

Figure 4 depicts the HRMS/MS spectrum for TP374 as well as the proposed fragmentation pattern. Fragment spectra were acquired alongside the mass voltammogram by DDA. The depicted spectrum was obtained by summation of HRMS/MS spectra with collision energies of 15 and 30 eV, respectively. The signal at *m*/*z* 356.1980 corresponds to the neutral loss of a water molecule supporting the previous assumption of TP374 being a hydroxylated product of MDMB‐4en‐PINACA. The signal at *m*/*z* 229.0979 can be explained through cleavage of the amide bond with the fragment ion still carrying the hydroxy group from the electrochemical hydroxylation. This suggests the hydroxy group being located in either the indazole core structure or the pentene side chain. Furthermore, the signal at *m*/*z* 230.1291 can be explained through an initial loss of the methyl formate moiety resulting in a fragment ion of *m*/*z* 314.1863. Again, the fragment ion still contains the hydroxy group supporting the previous thesis of localization. A following loss off the pentene side chain together with this hydroxy group results in the fragment ion of *m*/*z* 230.1288. Finally, the signal at *m*/*z* 290.1506 shows the loss of the pentene side chain together with the hydroxy group. Altogether, this confines the localization of the electrochemical hydroxylation to the pentene side chain.

**FIGURE 5:**
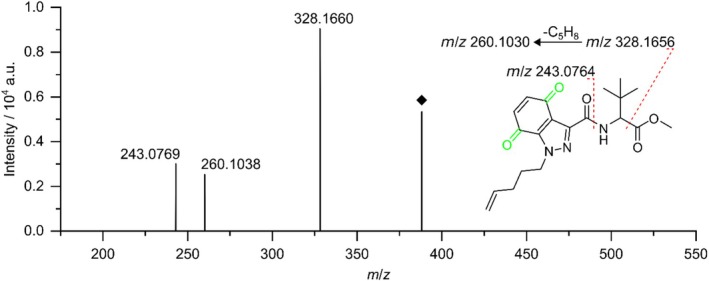
HRMS/MS spectrum and proposed fragmentation pattern of TP388. Changes in the molecular structure introduced during the electrochemical oxidation are highlighted in green. Because the absolute configuration of the TP is unknown, the fragmentation pattern is depicted based on the *para*‐quinone, although the formation of an *ortho*‐quinone would also be possible. The acquisition of fragment spectra was carried out alongside the mass voltammogram using DDA. The spectrum was obtained by summation of HRMS/MS spectra with collision energies of 15 and 30 eV, respectively.

Figure 5 depicts the HRMS/MS spectrum for TP388 as well as the proposed fragmentation pattern. Fragment spectra were acquired alongside the mass voltammogram by DDA. The depicted spectrum was obtained by summation of HRMS/MS spectra with collision energies of 15 and 30 eV, respectively. The signal at *m*/*z* 243.0769 corresponds to the loss of the amide side chain which is unchanged in comparison to the MDMB‐4en‐PINACA precursor structure. This suggests that the electrochemical modifications are either located in the indazole core structure or the pentene side chain. The signal at *m*/*z* 260.1038 further confines the localization of the electrochemical modifications. This signal can be explained through an initial loss of the methyl formate moiety resulting in a fragment ion of *m*/*z* 328.1656. A following loss of the pentene side chain results in the fragment ion of *m*/*z* 260.1030. The electrochemical modifications still being present in this fragment ion suggest that they are localized in the core indazole structure. Earlier, the mass difference of 29.9742 Da between TP388 and MDMB‐4en‐PINACA suggested that TP388 is a twofold hydroxylated and dehydrogenated product of MDMB‐4en‐PINACA. Together with the localization of these modifications in the indazole core structure, a quinoid structure can be proposed for TP388. This assumption is further supported by the absence of signals in the HRMS/MS spectrum that correlate to the neutral loss of water. Neutral loss of water would be expected if free hydroxy groups were formed but not in the case of the formation of a quinoid system. However, the absolute configuration of the quinoid system cannot be determined based on these data and therefore remains unclear.

**FIGURE 6:**
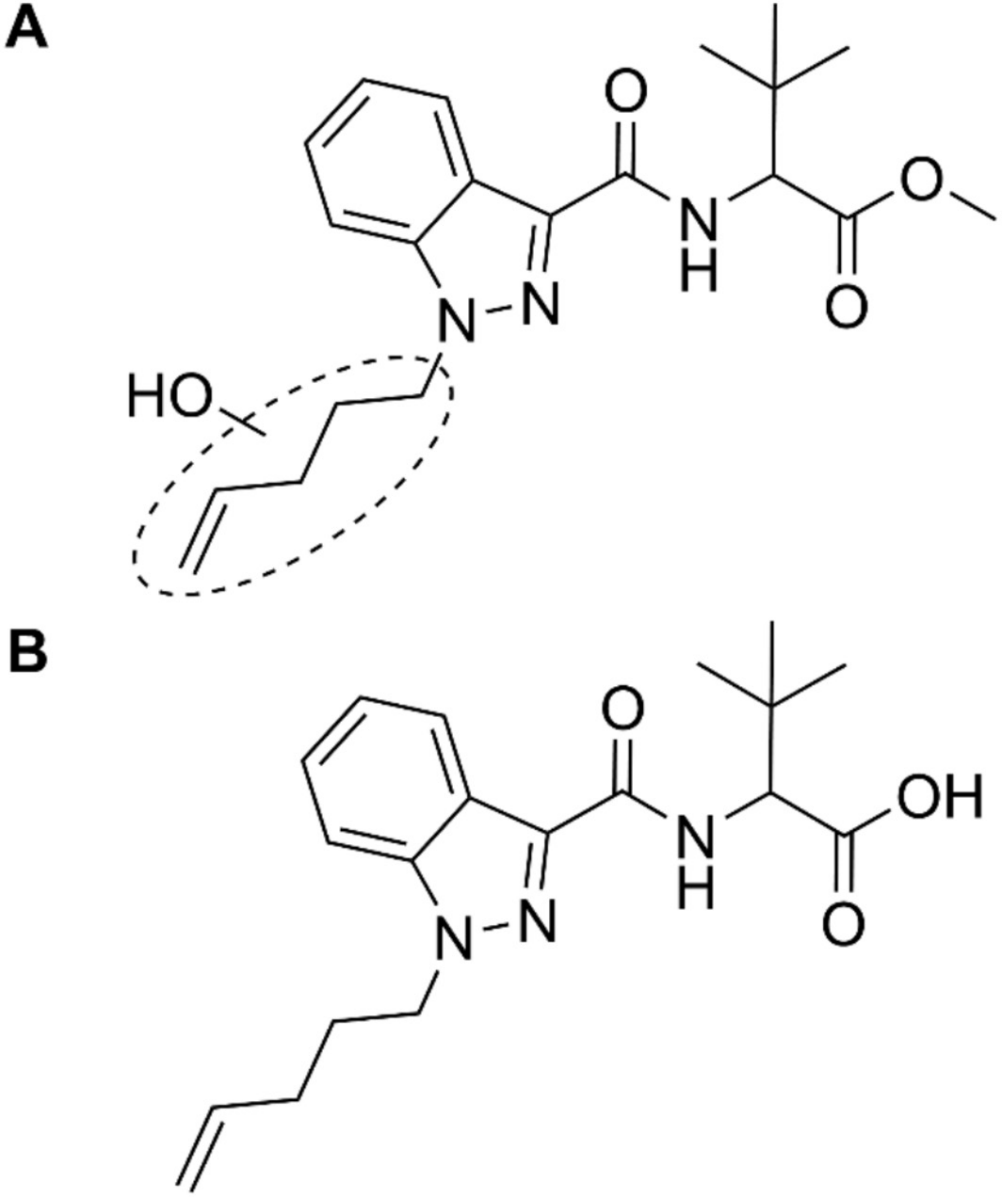
Molecular structures for a hydroxy metabolite (A) as well as a metabolite formed by ester hydrolysis (B) of MDMB‐4en‐PINACA. Both metabolites were recommended by Watanabe et al. as potential urinary markers for the intake of MDMB‐4en‐PINACA [30].

Watanabe et al. studied the metabolism of MDMB‐4en‐PINACA by incubating the drug with human hepatocytes as well as human liver microsomes and by analysis of authentic human in vivo blood and urine samples [30]. In total, they identified 32 different metabolites, two of which were found in the in vivo samples. Among these metabolites, two suggested urinary markers were a hydroxy metabolite as well as a metabolite formed by ester hydrolysis. Molecular structures for both metabolites are shown in Figure 6. On the one hand, the hydroxy metabolite found by Watanabe et al. likely corresponds to TP374 found in our study, demonstrating the ability of electrochemical metabolism simulation to form TPs which are also found as metabolites in conventional in vivo and in vitro studies. On the other hand, the metabolite formed by ester hydrolysis was not generated electrochemically. This is likely due to the ester hydrolysis not being a redox reaction, demonstrating the limitations of metabolism simulation by EC‐MS.

Furthermore, the electrochemically generated TP388 with a quinoid structure was not found by Watanabe et al. Because of the high redox activity of most quinones, a reaction of TP388 with endogenous molecules like peptides and proteins would be very likely in case of an in vivo formation of TP388. This could be a possible reason for the absence of TP388 as a metabolite in the work of Watanabe et al. In our electrochemical metabolism simulation, the well‐controlled chemical environment and absence of endogenous molecules prevent such reactions enabling the detection of highly reactive products like quinones. However, further conventional metabolism studies looking specifically for possible adducts of TP388 and endogenous molecules are needed to confirm the in vivo formation of TP388 as a metabolite. This is of interest because quinoid metabolites are known to induce severe oxidative stress in cells by oxidation of cellular macromolecules, thus causing acute cytotoxicity [31]. Therefore, the possible formation of TP388 as an in vivo metabolite could lead to the adverse and toxic side effects observed after the recreational use of MDMB‐4en‐PINACA [14].

## 4 Conclusion

In this study, a novel software tool for processing and evaluation of data acquired by EC‐MS experiments was developed. The software tool is based on and also fully incorporated into the open‐source mass‐spectrometry data evaluation software mzmine. Using this software tool, it is possible to generate interactive mass voltammograms from the mass spectrometric raw data as well as from processed feature lists. Furthermore, it is possible to reduce the spectral complexity of the generated mass voltammograms by utilizing the built‐in filtering algorithms in mzmine. Together, this greatly facilitates the evaluation process of data acquired by EC‐MS experiments and streamlines the process of metabolism simulation by EC‐MS.

Following this, the capabilities of the software tool were demonstrated by simulating the metabolism of the SCRA MDMB‐4en‐PINACA by EC‐MS from a mixed standard solution without the need for a pure reference standard of MDMB‐4en‐PINACA. This way, two main TPs were detected and their structures were tentatively elucidated using the acquired HRMS/MS data. The first TP was found to correspond to a hydroxy metabolite also found by Watanabe et al. in in vitro metabolism simulation of MDMB‐4en‐PINACA [30]. For the second TP, the HRMS/MS data suggested a quinoid structure. This TP was not found by Watanabe et al. as an in vivo metabolite, which might be a result of the high reactivity of most quinones. Because of this, a reaction of this TP with endogenous molecules like proteins and peptides would be possible, preventing its direct detection in human biofluids. Here, further conventional metabolism studies looking specifically for possible adducts of this TP and endogenous molecules are needed to confirm its in vivo formation as a metabolite. However, because quinoid metabolites are known to induce severe oxidative stress into cells, this could be a first hint into explaining the adverse and toxic side effects observed after the recreational use of MDMB‐4en‐PINACA [12, 13, 14, 31].

## Conflicts of Interest

The authors declare no conflicts of interest.

## Supporting information


**Table S1:** Mass spectrometric parameters used for the detection of TPs after metabolism simulation by EC‐HRMS/MS.
**Table S2:** Exact parameters used for DDA of fragment spectra of TPs generated by metabolism simulation utilizing EC‐HRMS/MS.

## Data Availability

The data that support the findings of this study are available from the corresponding author upon reasonable request.

## References

[1] M. Collins , “Some New Psychoactive Substances: Precursor Chemicals and Synthesis‐Driven End‐Products,” Drug Testing and Analysis 3, no. 7–8 (2011): 404–416, 10.1002/dta.315. 21755608

[2] European Drug Agency 2025. “European Drug Report 2025: Trends and Developments Luxembourg Publications Office of the European Union” https://www.euda.europa.eu/publications/european‐drug‐report/2025_en.

[3] F. Pantano , S. Graziano , R. Pacifici , F. P. Busardò , and S. Pichini , “New Psychoactive Substances: A Matter of Time,” Current Neuropharmacology 17, no. 9 (2019): 818–822, 10.2174/1570159X1709190729101751. 31577198 PMC7052837

[4] M. H. Baumann , E. Solis , L. R. Watterson , J. A. Marusich , W. E. Fantegrossi , and J. L. Wiley , “Baths Salts, Spice, and Related Designer Drugs: The Science Behind the Headlines,” Journal of Neuroscience 34, no. 46 (2014): 15150–15158, 10.1523/JNEUROSCI.3223-14.2014. 25392483 PMC4228124

[5] A. Di Trana , D. Berardinelli , E. Montanari , et al., “Molecular Insights and Clinical Outcomes of Drugs of Abuse Adulteration: New Trends and New Psychoactive Substances,” International Journal of Molecular Sciences 23, no. 23 (2022): 14619, 10.3390/ijms232314619. 36498947 PMC9739917

[6] F. Vincenti , C. Montesano , F. Di Ottavio , et al., “Molecular Networking: A Useful Tool for the Identification of New Psychoactive Substances in Seizures by LC‐HRMS,” Frontiers in Chemistry 8 (2020): 572952, 10.3389/fchem.2020.572952. 33324608 PMC7723841

[7] P. Hehet , N. Köke , D. Zahn , et al., “Synthetic Cannabinoid Receptor Agonists and Their Human Metabolites in Sewage Water: Stability Assessment and Identification of Transformation Products,” Drug Testing and Analysis 13, no. 10 (2021): 1758–1767, 10.1002/dta.3129. 34272823

[8] B. Pulver , S. Fischmann , F. Westphal , et al., “The ADEBAR Project: European and International Provision of Analytical Data From Structure Elucidation and Analytical Characterization of NPS,” Drug Testing and Analysis 14, no. 8 (2022): 1491–1502, 10.1002/dta.3280. 35524160

[9] V. Auwärter , S. Dresen , W. Weinmann , M. Müller , M. Pütz , and N. Ferreirós , “'Spice' and Other Herbal Blends: Harmless Incense or Cannabinoid Designer Drugs?,” Journal of Mass Spectrometry 44, no. 5 (2009): 832–837, 10.1002/jms.1558. 19189348

[10] World Health Organization 2020. “Critical Review Report: MDMB‐4en‐PINACA” https://cdn.who.int/media/docs/default‐source/controlled‐substances/43rd‐ecdd/mdmb‐4en‐pinaca‐review‐2020_b19597c3‐ac7e‐44bc‐b4db‐a40150a428c8.pdf.

[11] Bundeskriminalamt 2020–2024 “Statistisches Analyseprogramm NPS”.

[12] P. E. Oomen , D. Schori , K. Tögel‐Lins , et al., “Cannabis Adulterated With the Synthetic Cannabinoid Receptor Agonist MDMB‐4en‐PINACA and the Role of European Drug Checking Services,” International Journal on Drug Policy 100 (2022): 103493, 10.1016/j.drugpo.2021.103493. 34687992

[13] M. C. Monti , J. Zeugin , K. Koch , N. Milenkovic , E. Scheurer , and K. Mercer‐Chalmers‐Bender , “Adulteration of Low‐Delta‐9‐Tetrahydrocannabinol Products With Synthetic Cannabinoids: Results From Drug Checking Services,” Drug Testing and Analysis 14, no. 6 (2022): 1026–1039, 10.1002/dta.3220. 34997693 PMC9305195

[14] European Monitoring Centre for Drugs and Drug Addiction 2021. “European Drug Report: Trends and Developments Luxembourg Publications Office of the European Union” https://www.euda.europa.eu/edr2021_en.

[15] A. H. Lewin , H. H. Seltzman , F. I. Carroll , S. W. Mascarella , and P. A. Reddy , “Emergence and Properties of Spice and Bath Salts: A Medicinal Chemistry Perspective,” Life Sciences 97, no. 1 (2014): 9–19, 10.1016/j.lfs.2013.09.026. 24113072

[16] C. Norman , G. Walker , B. McKirdy , et al., “Detection and Quantitation of Synthetic Cannabinoid Receptor Agonists in Infused Papers From Prisons in a Constantly Evolving Illicit Market,” Drug Testing and Analysis 12, no. 4 (2020): 538–554, 10.1002/dta.2767. 31944624

[17] C. Norman , S. Halter , B. Haschimi , et al., “A Transnational Perspective on the Evolution of the Synthetic Cannabinoid Receptor Agonists Market: Comparing Prison and General Populations,” Drug Testing and Analysis 13, no. 4 (2021): 841–852, 10.1002/dta.3002. 33463894

[18] W. Lohmann and U. Karst , “Biomimetic Modeling of Oxidative Drug Metabolism: Strategies, Advantages and Limitations,” Analytical and Bioanalytical Chemistry 391, no. 1 (2008): 79–96, 10.1007/s00216-007-1794-x. 18163163

[19] A. Baumann and U. Karst , “Online Electrochemistry/Mass Spectrometry in Drug Metabolism Studies: Principles and Applications,” Expert Opinion on Drug Metabolism & Toxicology 6, no. 6 (2010): 715–731, 10.1517/17425251003713527. 20370599

[20] H. Faber , M. Vogel , and U. Karst , “Electrochemistry/Mass Spectrometry as a Tool in Metabolism Studies‐A Review,” Analytica Chimica Acta 834 (2014): 9–21, 10.1016/j.aca.2014.05.017. 24928240

[21] T. Herl and F.‐M. Matysik , “Recent Developments in Electrochemistry–Mass Spectrometry,” ChemElectroChem 7, no. 12 (2020): 2498–2512, 10.1002/celc.202000442.

[22] P. Fasinu , P. J. Bouic , and B. Rosenkranz , “Liver‐Based In Vitro Technologies for Drug Biotransformation Studies—A Review,” Current Drug Metabolism 13, no. 2 (2012): 215–224, 10.2174/138920012798918426. 22300020

[23] U. Jurva , H. V. Wikström , L. Weidolf , and A. P. Bruins , “Comparison Between Electrochemistry/Mass Spectrometry and Cytochrome P450 Catalyzed Oxidation Reactions,” Rapid Communications in Mass Spectrometry 17, no. 8 (2003): 800–810, 10.1002/rcm.978. 12672134

[24] H. Oberacher , F. Pitterl , R. Erb , and S. Plattner , “Mass Spectrometric Methods for Monitoring Redox Processes in Electrochemical Cells,” Mass Spectrometry Reviews 34, no. 1 (2015): 64–92, 10.1002/mas.21409. 24338642 PMC4286209

[25] V. Göldner , J. Ulke , B. Kirchner , et al., “Electrochemistry‐Mass Spectrometry Bridging the Gap Between Suspect and Target Screening of Valsartan Transformation Products in Wastewater Treatment Plant Effluent,” Water Research 244 (2023): 120525, 10.1016/j.watres.2023.120525. 37669607

[26] E. Niehaves and U. Karst , “Mimicking the Reactivity of Drug Metabolites: Biomolecule Conjugation of an Electrochemically‐Generated, Reactive Oxidation Product of the Antibiotic Minocycline,” Journal of Pharmaceutical and Biomedical Analysis 257 (2025): 116710, 10.1016/j.jpba.2025.116710. 39879820

[27] R. Schmid , S. Heuckeroth , A. Korf , et al., “Integrative Analysis of Multimodal Mass Spectrometry Data in MZmine 3,” Nature Biotechnology 41, no. 4 (2023): 447–449, 10.1038/s41587-023-01690-2. PMC10496610 36859716

[28] Y. Verkh , M. Rozman , and M. Petrovic , “Extraction and Cleansing of Data for a Non‐Targeted Analysis of High‐Resolution Mass Spectrometry Data of Wastewater,” MethodsX 5 (2018): 395–402, 10.1016/j.mex.2018.04.008. 30050758 PMC6060077

[29] S. Machado , L. Barreiros , A. R. Graça , R. N. Páscoa , M. A. Segundo , and J. A. Lopes , “A Data Mining Tool for Untargeted Biomarkers Analysis: Grapes Ripening Application,” Chemometrics and Intelligent Laboratory Systems 233 (2023): 104745, 10.1016/j.chemolab.2022.104745.

[30] S. Watanabe , S. Vikingsson , A. Åstrand , H. Gréen , and R. Kronstrand , “Biotransformation of the New Synthetic Cannabinoid With an Alkene, MDMB‐4en‐PINACA, by Human Hepatocytes, Human Liver Microsomes, and Human Urine and Blood,” AAPS Journal 22, no. 1 (2019): 13, 10.1208/s12248-019-0381-3. 31848852

[31] J. L. Bolton , M. A. Trush , T. M. Penning , G. Dryhurst , and T. J. Monks , “Role of Quinones in Toxicology,” Chemical Research in Toxicology 13, no. 3 (2000): 135–160, 10.1021/tx9902082. 10725110

