# Supplementary material for: Simulation of the Metabolism of New Psychoactive Substances Using Electrochemistry‐Mass Spectrometry: Introducing an Innovative Software Tool for Rapid Data Evaluation
Source: Drug Test Anal. 2025 Nov 27;18(2):222–9. doi: 10.1002/dta.70006 (PMC12861597; doi:10.1002/dta.70006)
Supplement: Supplementary file 1 — Table S1: Mass spectrometric parameters used for the detection of TPs after metabolism simulation by EC‐HRMS/MS. Table S2: Exact parameters used for DDA of fragment spectra of TPs generated by metabolism simulation utilizing EC‐HRMS/MS. [file DTA-18-222-s001.docx]

**Supporting Information**

**Simulation of the metabolism of new psychoactive substances using electrochemistry-mass spectrometry: Introducing an innovative software tool for rapid data evaluation**

Mark Wesner^a^, Steffen Heuckeroth^a^, Michael Pütz^b^ and Uwe Karst^a^*

^a^ Institute of Inorganic and Analytical Chemistry, University of Münster, Corrensstraße 48, 48149 Münster, Germany

^b^ Federal Criminal Police Office, Forensic Science Institute, Äppelallee 45, 65203 Wiesbaden, Germany

* Corresponding author: [uk@uni-muenster.de](mailto:uk@uni-muenster.de)

# SI-1 Additional experimental details

Exact mass spectrometric parameters used for the detection of transformation products (TP) after metabolism simulation by electrochemistry coupled to high-resolution tandem mass spectrometry (EC-HRMS/MS) are listed in Table SI-1.1. Parameters used for data-dependent acquisition (DDA) of fragment spectra alongside the mass spectrometric detection are further listed in Table SI-1.2.

Table SI-1.1: Mass spectrometric parameters used for the detection of TPs after metabolism simulation by EC-HRMS/MS.

| **Parameter** | **Description** |
| --- | --- |
| **Ionization mode** | Positive ion electrospray |
| **Spectra Rate** | 4 Hz |
| **Source** |  |
| **End Plate Offset** | 500 V |
| **Capillary** | 3500 V |
| **Nebulizer** | 0.3 bar |
| **Dry Gas** | 4 l min^-1^ |
| **Dry Temp** | 200 °C |
| **Tune** |  |
| **Deflection 1 Delta** | 70 V |
| **Funnel 1 RF** | 250 V_pp_ |
| **isCID energy** | 0 eV |
| **Funnel 2 RF** | 500 V_pp_ |
| **Multipole RF** | 450 V_pp_ |
| **Collision Energy** | 5 eV |
| **Collision RF** | 550 V_pp_ |
| **Ion Energy** | 5 eV |
| **Low Mass** | 50 *m*/*z* |
| **Transfer Time** | 50 µs |
| **Pre Puls Storage** | 13 µs |

Table SI-1.2: Exact parameters used for DDA of fragment spectra of TPs generated by metabolism simulation utilizing EC-HRMS/MS.

| **Parameter** | **Description** |
| --- | --- |
| **MS/MS Spectra** |  |
| **Rate Control** | Same as MS spectrum |
| **Multi CE** | 50% & 100% |
| **MS Filter** |  |
| **Normalized Threshold** | 31 counts/1000 scans |
| **Precursor Ion Selection** |  |
| **Selection by** | Fixed number |
| **Number of Precursors** | 3 |
| **Active Exclusion** |  |
| **Exclude after** | 5 spectra |
| **Release after** | 1 min |
| **Isolation & Fragmentation** |  |
| **Isolation Width** | 1 *m*/*z* |
| **CE** | 30 eV |
| **Fallback Charge State** | 1 *z* |
